# Improving complex systems with improve-mentation: challenges and solutions

**DOI:** 10.3389/frhs.2025.1724893

**Published:** 2026-01-20

**Authors:** John Ovretveit

**Affiliations:** Department of Learning, Informatics, Management and Ethics, Karolinska Institutet Medical University, Stockholm, Sweden

**Keywords:** implementation, improvement, complexity, adaptive implementation, context, quality methods, implementation strategies, implementation tools

## Abstract

The fast-changing environment for healthcare in all countries calls for new approaches to achieving improvements. This article proposes that improve-mentation is one such approach. Improve-mentation synergistically combines elements of implementation and improvement sciences, as well as experience to carry out change in different settings. One feature is the iteration of the change so as to adapt the change to the evolving context for private and public healthcare in different countries. The article addresses challenges posed by increasing complexity and describes the methods used in four different improve-mentation frameworks, using case examples to illustrate different resolutions to generalisability and other issues.

## Introduction

This article addresses the problem of how improvement research and practice can be modified to respond more effectively to the new and fast-changing healthcare environment by integrating elements of implementation science and practice. Reed et al. (1) have provided a comprehensive overview of improvement science and of recent developments. This article describes one approach, termed “improve-mentation”, and how it can help meet the current challenges, not the least of which is how to integrate Artificial Intelligence (AI) safely to achieve improvements.

Improve-mentation combines the elements of improvement and implementation sciences as appropriate to the problem and setting to achieve better healthcare and outcomes through research-informed, effective and timely change. The approach has been used to apply evidence-based medicine (EBM), defined as “the process of systematically finding, appraising and using contemporaneous research findings as the basis for clinical decisions” (2). Evidence-based healthcare (EBHc) is a broader concept including evidence-based management and policy, often defined as using evidence to inform actions for groups of patients or populations, although the concept can also apply to clinical decisions about individual patients.

Implementation of EBM and EBHc in healthcare has been challenging and so has sustaining and scaling up proven interventions. Disseminating information about the content of a proven effective clinical intervention alone does not seem to be effective (3). Telling clinicians or other decision-makers what an evidence-based (EB) intervention achieves—i.e. the content of the intervention—does not, on its own, change practice (4). Evidence of efficacy from a trial in one setting is not evidence of effectiveness in all other settings (5). For example, most evidence of improvements for hand hygiene is from research carried out in hospitals, but there is little evidence from different primary care settings or many low-resource settings. In part, this is because biochemical drug treatments have similar effects on human bodies, but social interventions will have significantly different effects in different social and organisational settings.

## Complexity

Research has shown that accomplishing most changes in healthcare is difficult, costly and time-consuming (6). Even ensuring effective handwashing is challenging. One explanation is that the intervention content involves multiple changes: All staff and visitors need to use alcohol-based hand sanitiser at specific times and change other behaviours, and implementation requires effective training, ensuring sanitiser is always conveniently available where needed, and other changes.

### Complexity of clinical practice and healthcare

Research suggests that the complexity of clinical practice and healthcare is one of the explanations for this difficulty: Different professions and staff often each have to make changes to their daily practice, and these need to be coordinated to be effective. Usually, changes to health information technology and other changes are needed to support clinical practice (7).

### Complexity of the innovation

Many proven innovations can also be considered complex. One example is sepsis care “bundles” referring to the multiple parts of the intervention (8, 9).

### Complexity of the implementation actions

Not only can innovations and healthcare be considered as complex but also can the implementation action steps. For example, implementation actions that were necessary to change clinical practice to improve outcomes were reported for one clinic for women veterans (41). This empirical description reports multiple action steps in sequence and in parallel under four categories of actions: stakeholder engagement carried out at different levels of the health system, data-based progress monitoring, adaptation, adjustment and sharing results. Related to the implementation actions are the different values of actors and stakeholders who need to take part in the implementation, summarised in the concept of “value complexity”: “the complexity arising from differences in people's world-views, interests and values, leading to mistrust, misunderstanding and conflict among stakeholders” (10, 11).

There is certainly more research into barriers to implementing EBHc than empirical research into the details of successful implementation, and even less into successful sustainment and scale-up of an EBHc innovation over time and different settings (12). Our narrative review of the research into implementation of EBHc innovations (13, 14) found that the main gaps in knowledge that hinder implementers and researchers can be summarised as follows:

- a lack of detailed empirical knowledge about the decisions and activities taken by different parties to implement different EBHc innovations,
- limited understanding of which of these actions were most significant in helping or hindering the operation of the EBHc innovation in daily practice,
- knowledge about which features of the context of practice, the organisation and external factors such as the financing system and regulations affect the take-up of different EBHc innovations and which of these are most significant, and
- knowledge about how implementers and researchers can use or not use research into one implementation of an EBHc innovation to understand implementation in another setting.

## History of implementation and improvement science

Historically, researchers and practitioners of each science have remained as separate tribes and communicated little, although this is now changing to some degree. One difference between improvement and implementation sciences is that each originated in different settings and is oriented to different purposes. Improvement methods originated in the manufacturing industry, and theories and empirical research in healthcare were later developed. Implementation science had a more academic and recent origin. In healthcare, it was driven by a concern about delays in implementing EBM and findings from research. Its development emphasised using research-informed methods and theories from organisation and behaviour and other sciences to formulate the practical methods, tools, and strategies (15). Some of these differences are summarised in Table 1.

**Table 1:** Differences between improvement and implementation sciences in healthcare in high-resource settings.

Improvement science	Implementation science
The ideas for the improvement changes to be made can come from different sources	Focus on tested EB practices implementation
Usually, the idea for change is iteratively adapted when making the change	Exact copies of tested EB practices were the original focus
Originated in the manufacturing industry with a pragmatic objective	In healthcare, motivated by “delay” in implementing tested EB practices and guidelines
Scientific research to explain and evaluate the methods, projects or programmes came later. Most of the earlier theory was experience-based (e.g. the Deming system of profound knowledge) (40)	Scientific research came first, and an implementer occupation later developed
Practical tools that staff can use are well-developed and packaged.	Practical tools that staff can use are few and under-tested such as data collection methods
Large community of practitioners and a smaller research community	Less well known in healthcare than improvement science. Large community of researchers
Mostly practice-based	Mostly an academic or consulting base
Common to both is a purpose to improve healthcare and outcomes, and systems thinking	

EB, evidence-based.

## Rationale and need to combine the sciences

In many ways, the purposes are the same: to make healthcare better and improve outcomes for patients, using an applied science orientation. Recent developments in both sciences and practice have provided more understanding of the importance of “informal context” and the need to uphold certain values to motivate changes. Research on private equity ownership of healthcare has added to the understanding of the dissatisfaction of clinicians and challenges recruiting staff, which in some ways is a more extreme version of corporatisation and consolidation of all healthcare (42). This has led some healthcare organisations to change their culture to align more with clinicians' values as well as to change formal structures to adjust workloads (10, 11).

Conceptually, improvement science has evolved a convention to differentiate the intervention content from the implementation strategy, as this was found to be necessary to describe the implementation process. Traditionally, improvement science has not made this distinction, and reports have been criticised for not giving clear descriptions of what was implemented and how it was, which then makes generalisation of the findings difficult. The improvement science convention is to distinguish between the EBHc innovation, sometimes called the “content” of the intervention or “the thing” to be implemented, and the implementation actions taken to establish this innovation in routine healthcare operations in a particular setting (16). Implementation science has also found it necessary to describe the different actions taken at different levels that help and hinder implementation.

Other trends that align with the improve-mentation approach are towards co-design and co-production. Researchers and funders have recognised how both increase the value of research and of healthcare delivery. The iteration and feedback in co-design and co-production are an integral part of improve-mentation (17, 18). This also includes recent studies that operationalise co-design, adaptive measurement, and participatory evaluation. These models demonstrate how iterative cycles, stakeholder involvement, and dynamic evidence generation occur in practice across complex health systems (19, 20).

## Approaches to support improve-mentation in practice

Some publications remark on the overlap and suggest more cooperation between the two domains. In cancer care (21), note that, “Due to their relative isolation, implementation and improvement science have not realized potential synergies to drive positive change in delivery”. Earlier, in the same field (22), a “call to action” was published to “harness the synergy” between the two sciences. On the same theme, Ogrinc et al. (43) proposed that “QI can learn from IS by incorporating framework driven approaches to development, planning, and evaluating outcomes that are helpful to make the work more easily generalisable. IS can learn from QI by incorporating data driven flexibility, needed to show how interventions can be successful in a wide variety of contexts”.

A review by the author and colleagues found four formulated, coherent and applicable methods and strategies which combine elements of each science for different objectives. These four approaches and their purpose to support improvement are discussed below: the “integrated improve-mentation framework (IIF)”; the “learning evaluation” approach; the active implementation frameworks (AIFs) approach; and the “getting to outcome framework” approach.

## Example of an improve-mentation approach

To illustrate one approach, the following is an example from the first year of the COVID-19 emergency in Stockholm (23). In March 2020, when the first COVID-19 cases and deaths occurred, researchers from the Medical Management Center at Karolinska Medical University formed a joint improve-mentation team with leaders from the Stockholm healthcare organisation (23, 24). The researchers collected the emerging and scarce evidence about the virus and presented it regularly to the managers of the organisation, together with information about the degree of certainty of the evidence and comments about the relevance to action in Stockholm using the Agency for Healthcare Research and Quality (AHRQ) grade score (44). They also surveyed the 120 leaders of front-line facilities and summarised and presented this evidence to the management team and to the 120 leaders. Guidance and support were provided to leaders about improvement science methods and quality improvement (QI), such as local practical data collection and analysis, plan-do-study-act (PDSA) iteration cycles, and the improvement framework (45, 46). Moreover, support from implementation science (IS) about practical behaviour change and organisational change methods was provided (25–27).

### Features of improve-mentation in the illustration

Details about how guidance and support were provided to leaders are reported in previous studies (23, 24). There are points of note about this example that help explain this approach. First, most of the “evidence implemented” could not be described as proven EBHc interventions. The author remembers not touching mail deliveries for 3 days because we did not know if the virus could be spread on paper. Much of the evidence about the virus that we found and presented was “experiential evidence”, initially from our Italian colleagues who were the first to report and share their experience with internet video presentations and publications [e.g. (28)]. In some ways, this “evidence” was more useful and less misleading than the evidence in many of the scientific pre-print publications. Experience and expertise in both improvement and implementation research and sciences were needed to assess this, and not burden management with reports involving inconclusive or inactionable findings.

This “certainty of evidence” question is important for two related reasons. First, with low certainty evidence, the intervention or implementation strategy will need testing locally, using improvement science methods if time is short. Secondly, even with higher-certainty evidence from one setting, this evidence may not apply in another setting and will also need testing (47). We also note that the evidence base of many improvement bundle innovations is limited (29). However, this is less of a problem if the local implementation uses testing and iterative adaptation, for example, using improvement science methods (e.g. PDSA). Related to these points is another consideration: Staff learning and using improvement and implementation science and methods in project teams develop their and the organisation’s capacity to implement other changes. It helps build a more resilient learning organisation, more capable of responding to the changes demanded by changing technology and other contextual factors.

### Integrated improve-mentation framework

The COVID-19 example above described features of one of the four approaches to improve-mentation called the “integrated improve-mentation framework (IIF)” (15). The purpose that this approach serves to support improvement is to give researchers and practitioners a five-step framework they can follow that uses methods from both sciences in each step. The purpose of a later version is to show how to use digital technologies and data for the rapid cycle parts of the framework (23). The features were summarised as “(1) define problem; (2) decide data indicating problem solved; (3) design and implement solution; (4) review data and revise solution; and (5) repeat as situation changes” (15, 24, 48). The digital version emphasises focusing on outcomes attributable to the change where there is reliable digital data or where digital technologies can access these data. Examples are in improving prescribing for antibiotics, where rapid iteration of the change on small samples using statistical methods can help implementation.

### Learning evaluation

A second approach is for researchers and is termed “learning evaluation”. The purpose that this approach serves to support improvement is to give researchers a guide with an example of how they can contribute to improvement in their research role. It “blends quality improvement and implementation research methods to study healthcare innovations” (49). This is summarised as “Integrating implementation and evaluation of interventions by establishing feedback loops…that allow the intervention to adapt to ongoing contextual changes…”.

This study reports the approach using an example of a study of two mental health and nine primary care units. The units chose one type of evidence-based practice to implement to improve integration between behavioural health specialists and primary care practitioners and promote patient-centred care. In this approach, the researchers play a prominent role, and it should be noted that without the researchers to facilitate and carry out data collection, it may not be possible for practitioners or organisations to use the approach. Some organisations may have a well-developed improvement and implementation capacity with suitable data collection and analysis support to their implementation teams as well as teams with skills in carrying out PDSA and using other improvement methods.

The article gives an example of the approach in the 11 units where the researchers helped the practitioners to use PDSA and collected and reported back the data to the implementation teams so that each team could make adjustments. The approach is intended to be used flexibly, by following five principles in carrying out this type of action evaluation: “Others who may want to replicate this approach can use the general principles and adapt them to their specific contexts. Adhering to the principles can help a single system or a multi-site demonstration project collect data and report findings that are highly transportable and that also provide contextual understanding for others who wish to reinvent their interventions” (49). The following five principles, as applied, are described in further detail in the example given in the latter report:

1) Collect data: This included understanding the implementation plan; the data to describe the intervention change and implementation steps, as observed; and then baseline data to track progress in implementation.
2) Collect outcome data: Gather information about the implementation process and subsequent outcomes needed by both the research team and implementers in the units.
3) Collect data about context influences: Identify influences from different levels of the healthcare system that impact implementation actions and outcomes and that will help others to decide how they might make adaptations in different contexts.
4) Analyse and share data: Analyse the collected data and facilitate the implementers to use it for improvement and develop their systems to do this.
5) Operationalise the measurement methods and help others to learn from this implementation.

Finally, this article describing this approach characterises it as being relevant to the concept of a learning health system and as an adaptable approach to rigorous, relevant and fast evaluation (30–32).

### Active implementation frameworks

This approach encompasses five frameworks [described in McColskey-Leary & Garman-McClaine (50), and originally by Fixsen & Fixsen (51)]. The purpose that these five frameworks serve to support improvement is to give different but related models that both researchers and improvers can use to improve welfare services using elements of improvement and implementation science. The frameworks come from research and practice in implementing innovations in child and family welfare services and in other services. Articles and the website describe how to use the five frameworks (33). The article by Fixsen and Fixsen (51) and the web tools describe an equation for implementation success: effective innovations × effective implementation with enabling contexts = impactful outcomes.

### “Getting to outcome framework” (GTO)

The purpose that this approach serves to support improvement is to provide researchers and implementers with specific methods for ensuring accountability for the results of an improvement programme and to combine improvement and implementation sciences in different steps of the 10-step approach. This 10-step approach exists in different versions described for different implementations, including complex, large-scale implementations (34–36). Other examples include illness prevention (37); a hand hygiene evidence based practice (EBP) implemented in a low-income setting providing excellent results (52); and telephone-based mental health services for underserved communities in the USA (38). In relation to these versions, Wandersman et al. (39) elaborated on the facilitation support needed and described the practical tools, including the improvement cycle, in their Figure 2 (39).

With the rapid increase in data-rich clinical and management environments, we are entering a new era where improve-mentation developments may become more relevant. Recent developments in learning health systems and in tools and methods for automated learning loops, Bayesian decision processes, and context-aware feedback can be incorporated into improve-mentation (19, 32).

## Conclusion

Improve-mentation is an approach that synergistically combines elements of the implementation and improvement sciences as well as experience in carrying out change in different settings. Part of what many refer to as “the delay” in healthcare in adopting proven improvements and innovations into everyday practice is due to the complexity of healthcare, innovations, and implementation actions needed. Improve-mentation addresses the complexity of healthcare by assessing which influences at different levels of the health system help and hinder the innovation and by using quality and implementation tools to adapt and test the changes made so as to adjust to the changing context.

This article proposed that an improve-mentation approach can provide practical guidance and scientific knowledge for resolving some of the challenges of complexity, especially in the new era of AI. Such an approach combines methods and knowledge from both implementation and improvement sciences and practice to enable change in healthcare and implementation of EBHc. The four different approaches share five distinctive features of improve-mentation, including (1) pre-implementation frameworks to guide data collection, (2) understanding contextual factors that help or hinder the innovation’s effectiveness and its implementation, (3) documentation and measurement of outcomes, (4) iterative small-scale testing and feedback, and (5) close collaboration between investigators and implementers.

## Data Availability

Publicly available datasets were analysed in this study. These data can be found here: narrative review.
